# Annotations

**Published:** 1899-01-14

**Authors:** 


### ANNOTATIONS.

### The Health of London.

THE reports of medical officers of health, crammed as they are with figures and tabulations, are not usually attractive to the general reader. In the report of the medical officer of health of the County of London which has recently been issued there are, however, many points which are of much general interest. First, as regards the boasted healthiness of London. No doubt, taking the death-rate of the whole of London and comparing it with that of other towns we come off pretty well, having had in the year 1897 a lower death-rate than any other English town with a population of 200,000 and over, with the exception of Bristol and West Ham. But when we come to divide London into districts, and inquire into the death-rates which obtain in them, we find that the variations are very great, so that, while in Hampstead, Lewisham, and Plumstead they were from 13·3 to 13·7 per thousand, they ran, on the other hand, between 27·3 and 27·8 in Limehouse, St. George's-in-the-East, and St. Luke's, being twice as high in one group of districts as in the other; so that, when people say that London is a healthy place, we must always ask what part of London they mean. It is when we come to compare London with other capitals of Europe in regard to deaths from zymotic disease that we, a professed nation of sanitarians, must feel that our pride sustains a fall. Taking the six principal zymotic diseases—namely, small-pox, measles, scarlet fever, diphtheria, whooping-cough, and fever—we find that the death-rate in London was 1·68 per 1,000 living. Compared with many of our provincial towns this is fairly good; but compared with Paris, 0·68; Brussels, 0·51; Berlin, 0·88; and Rome, 0·65, it is very bad, indeed, and serves to show how quickly we are being overtaken in the race for health. Again we find the extremest differences between the incidence of this zymotic death-rate on different parishes; for, while in St. George's, Hanover Square, it stands at 1·10, in St. George's, Southwark, and in Bermondsey it reaches 4·35 per 1,000 persons living; very nearly four times as great a mortality from this group of diseases in some parishes as in others.

### "Nursing Streets."

IT is not entirely without reason that the residents in certain districts in the West End object to houses in their streets being turned into nursing homes. Not that we need have any sympathy with the more or less sentimental objections which are sometimes raised to the continued propinquity of sick people. Still, when houses are close together the sick are of necessity a nuisance to the healthy. That one's piano should be hushed, that sounds of revelry by night should not be permitted to arise, and that one should live in a state of perpetual consideration for the feelings of one's neighbour, are things which, although distinctly disagreeable, one puts up with with such grace as may be possible when a *bonâ-fide* neighbour is seriously ill. We do for others what we expect to be done for us. The matter, however, is rather different when a constant series of sick people are imported into a street for purely business purposes. It is often said that people who live in London know nothing about their next-door neighbours. In a sense, that is true; they often, nay generally, do not "know" them in a society sense, but it is impossible not to know a good deal that is going on in a street in which one lives. There is a backstairs freemasonry, and through the maids things leak out, and although one, no doubt, has a perfect right to bang one's doors, or to take one's dog a run, yapping all down the street, before turning in at night, or even to entertain one's guests with music as music is now sung and played, still the rumour that someone in the next house is now just at the crisis of his life, that some grave operation has been done, and that all depends on sleep; all this sort of thing, while not touching the legality of our little joys, takes off the cream. When this goes on from year to year, we need not wonder that nursing homes are not very popular in residential quarters. There is, moreover, another aspect of the matter to which attention has recently been called by the medical officer of health for Marylebone, namely, the great frequency with which "nursing streets" are covered with litter for the sake of ensuring quietness. He recently presented a report on the unsatisfactory state of the law on the subject, and said that "it could hardly have been contemplated to give any person power to lay down litter without sanction, . . . In any case it will be far more satisfactory to obtain, if possible, definite powers to make regulations as to the deposition of straw." And he adds: "The frequent complaints about this straw-laying sufficiently show that it is becoming a nuisance of some magnitude; that what may be called the nursing streets are almost permanently covered with straw; and that apparently there do not exist sufficiently definite powers to deal with the litter or straw until it becomes offensive." Here, again, one does not protest even against a nuisance when it comes once in a way in the course of ordinary illness, and when it is for the benefit of *bonâ-fide* neighbours. But to live in a straw-yard—and such a straw-yard as a London street becomes after a couple of days—for the sake of unknown people, imported for the profit of an institution, is more than average good nature is likely to put up with.

### Proving Too Much.

THERE seems every reason to believe that Haffkine's inoculation has some influence in rendering people, for a time at any rate, more or less immune to plague. It only "stands to reason," if we may use such a phrase, that this should be so. That a man, when inoculated with a modified virus of such a malady as plague, should attain a certain degree of immunity to the major disease is perfectly analogous, not only with vaccination, but with all that we know about the conduct of similar infections when inoculated into the lower animals; and from the statistics which are given as to the results it seems to be the fact that a protective influence against plague has really been exerted on those who have subjected themselves to the process. It seems, then, a pity to spoil everything by claiming too much. When we find it gravely stated in a leading medical journal that, "besides the prophylactic effect against plague, the inoculation would seem to have a good result in cases of chronic malarial fever," we cannot but feel that this new appendix to the inoculation creed is rather too severe a strain upon our faith.

